# A large deletion in *RPGR* causes XLPRA in Weimaraner dogs

**DOI:** 10.1186/s40575-016-0037-x

**Published:** 2016-07-08

**Authors:** Regina Kropatsch, Denis A. Akkad, Matthias Frank, Carsten Rosenhagen, Janine Altmüller, Peter Nürnberg, Jörg T. Epplen, Gabriele Dekomien

**Affiliations:** Department of Human Genetics, Ruhr-University, Universitätsstraße 150, 44801 Bochum, Germany; Kleintierklinik Frank, Mooswaldallee 10 i, 79108 Freiburg, Germany; Tierärztliche Überweisungspraxis, Lortzingstraße 12, 28209 Bremen, Germany; Cologne Center for Genomics, University of Cologne, Weyertal 115 b, 50931 Cologne, Germany; Institute of Human Genetics, University of Cologne, Kerpener Str. 34, 50931 Cologne, Germany; Center for Molecular Medicine Cologne (CMMC), University of Cologne, Robert-Koch Str. 21, 50931 Cologne, Germany; Cologne Excellence Cluster on Cellular Stress Responses in Aging-Associated Diseases (CECAD), University of Cologne, Joseph-Stelzmann-Str. 26, 50931 Cologne, Germany; Faculty of Health, University Witten-Herdecke, Alfred-Herrhausen-Strasse 50, 58448 Witten, Germany

**Keywords:** Progressive retinal atrophy, Exome sequencing, *RPGR* (retinitis pigmentosa GTPase regulator) gene, Weimaraner

## Abstract

**Background:**

Progressive retinal atrophy (PRA) belongs to a group of inherited retinal disorders associated with gradual vision impairment due to degeneration of retinal photoreceptors in various dog breeds. PRA is highly heterogeneous, with autosomal dominant, recessive or X-linked modes of inheritance. In this study we used exome sequencing to investigate the molecular genetic basis of a new type of PRA, which occurred spontaneously in a litter of German short-hair Weimaraner dogs.

**Results:**

Whole exome sequencing in two PRA-affected Weimaraner dogs identified a large deletion comprising the first four exons of the X-linked retinitis pigmentosa GTPase regulator *(RPGR)* gene known to be involved in human retinitis pigmentosa and canine PRA. Screening of 16 individuals in the corresponding pedigree of short-hair Weimaraners by qPCR, verified the deletion in hemizygous or heterozygous state in one male and six female dogs, respectively. The mutation was absent in 88 additional unrelated Weimaraners. The deletion was not detectable in the parents of one older female which transmitted the mutation to her offspring, indicating that the *RPGR* deletion represents a *de novo* mutation concerning only recent generations of the Weimaraner breed in Germany.

**Conclusion:**

Our results demonstrate the value of an existing DNA biobank combined with exome sequencing to identify the underlying genetic cause of a spontaneously occurring inherited disease. Identification of the genetic cause has allowed the development of a diagnostic test, which should help to eradicate the PRA causing mutation from the respective canine line. Thus, planning of future pairings is facilitated and manifestation of this type of PRA can be prevented.

**Electronic supplementary material:**

The online version of this article (doi:10.1186/s40575-016-0037-x) contains supplementary material, which is available to authorized users.

## Plain English Summary

Progressive retinal atrophy (PRA) affects cats and dogs, initially causing night blindness, followed by gradual visual loss and finally blindness. PRA comprises a large group of genetically heterogeneous retinal diseases with similar clinical appearance but differing in age of onset and rate of disease progression. Mutations in more than 20 genes are known to cause canine PRA, but the genetic cause of PRA in several affected breeds still remains to be identified. Most forms of canine PRA are inherited in an autosomal recessive manner, caused by mutations in the same gene transmitted from both parents. Autosomal dominant inheritance, in which one mutation suffices to cause the disease, is rarely observed. Three X-linked forms have been found to be caused by different mutations in the canine retinitis pigmentosa GTPase regulator (*RPGR*) gene.

Since a new type of PRA was diagnosed in a single litter of Weimaraner dogs in March 2015, we investigated all genes of two affected males in comparison with healthy controls. We analysed the DNA of all coding parts of the genes (known as exons) using exome sequencing analysis. A large gap was identified in the *RPGR* gene, *i.e.* a deletion comprising the first four exons. The X-linked *RPGR* gene is known to be involved in both human retinitis pigmentosa and canine PRA. Screening of affected and healthy Weimaraners revealed the new mutation in hemizygous state in one affected male and in heterozygous state in six mildly affected or asymptomatic female dogs, whereas it was absent in all unrelated Weimaraners investigated. The deletion was not present in the parents of one older female which transmitted the mutation to her offspring. This indicates that the *RPGR* gene was newly mutated in this female or in the parental sperm or egg, respectively. The established DNA test allows easy mutation detection and helps the breeder community to plan future pairings for the next generations of Weimaraners without this type of PRA.

## Background

Retinal dystrophies are a common cause of blindness in both human beings and purebred dogs (*Canis familiaris*). The most typical form of canine dystrophy is PRA which is an equivalent in phenotype and disease progression to retinitis pigmentosa (RP) in man [1]. PRA encompasses a large group of genetically heterogeneous retinal dystrophies that share a similar phenotype of progressive visual impairment, finally leading to blindness. Initially, rod photoreceptor vision is affected resulting in night blindness, followed by progressive loss of cone photoreceptors with deterioration in daytime vision [2]. In other forms of retinal diseases such as cone-rod dystrophy and achromatopsia, daytime vision is impaired prior to night vision [3]. PRA has been documented in more than 100 different dog breeds [2]. To date, mutations in at least 20 genes have been associated with PRA [4]. However, the genetic cause of PRA in several affected breeds still remains to be identified. Most forms of canine PRA are inherited in an autosomal recessive manner, but an autosomal dominant trait [5] as well as three X-linked forms are also known [6–8] which arose independently as single mutation events [9]. The different forms of PRA can be classified by the age of onset of night blindness and the rate of disease progression (i.e., vision loss) [2].

In Germany, in March 2015, several litter mates of Weimaraners (*Fédération Cynologique Internationale* group 7, section 1, standard No. 99) aged ~2.5 years were ophthalmologically diagnosed with a bilateral retinal dystrophy resembling PRA. The Weimaraner, originally designated as Weimar pointer, represents a native old German breed of hunting dogs. Its origin can be traced back to the 18th century when the first breed standard was established [10]. With about 500 puppies per year in Germany (http://www.vdh.de/ueber-den-vdh/welpenstatistik/; *Verband für das Deutsche Hundewesen*, VDH) the Weimaraner does not have a particularly small population base. Hence, the molecular genetic basis of PRA in this breed was studied using next generation sequencing (NGS). A large deletion was identified in the *RPGR* gene, mutations of which have been reported to cause X-linked RP (XLRP) in man [11] and X-linked PRA in dogs [7].

## Methods

### Dogs

**Fig. 1:**
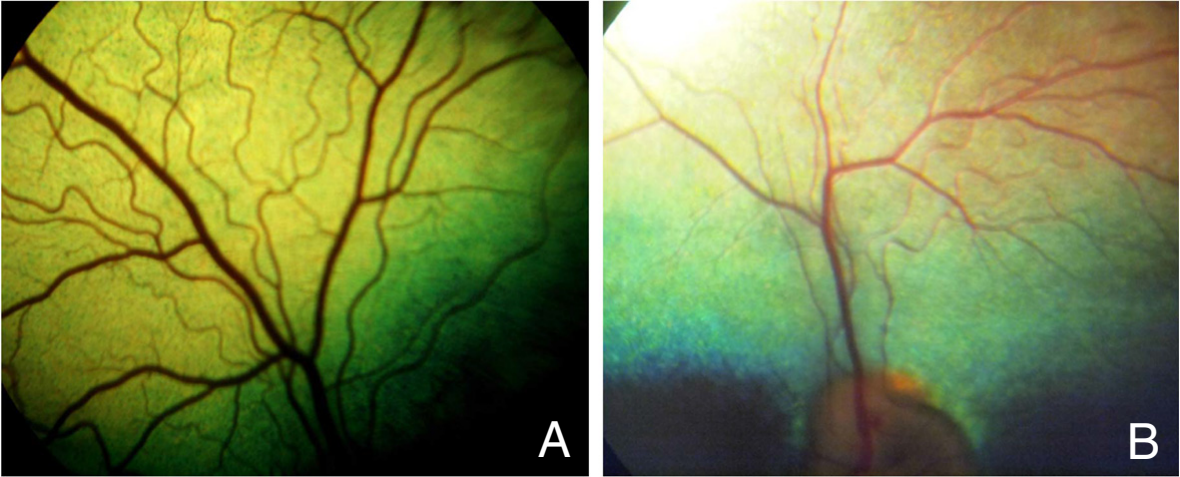
Ophthalmoscopic appearance of the central fundus. **a** Healthy unrelated female Weimaraner dog (~3 years) with a normally appearing fundus (right eye). **b** PRA-affected male Weimaraner dog (~2.5 years) showing the typical landmarks of PRA with vessel attenuation primarily of the arterioles in the papillary area and a hyperreflective *tapetum lucidum* (left eye)

All investigated dogs originated from the common breeding population of pure-bred short-hair Weimaraners. Veterinarians who were specialized and experienced in ophthalmological diseases (*Dortmunder Kreis*, DOK) confirmed the PRA status of affected male dogs (*n =* 3; Fig. 1b) and of female dogs with mild symptoms (*n =* 3) by ophthalmoscopy, as documented in certificates of eye examinations. For all other dogs, general veterinarian examinations revealed normal sight as prerequisite for breeding. In total, blood samples of 108 dogs were received from the owners either submitted to the Weimaraner DNA biobank (established at the Department of Human Genetics, Ruhr-University Bochum, Germany) or sent to our institute for other research projects such as investigation of genetic variability in the Weimaraner population. Written informed consent was obtained from the owners for sample collection and genetic investigations. Sample collection was performed by practicing veterinarians according to international guidelines for the use of laboratory animals. As the DNA stems from the blood of client-owned dogs that underwent routine veterinary examinations including venipuncture, animal experiments were not performed and, therefore,approval by an ethical committee was not necessary. Genomic DNA was isolated from peripheral blood cells using a standard protocol [12]. To test matrilineal inheritance of PRA in Weimaraners, mitochondrial genome sequencing was performed in one PRA-affected dog and his mother.

**Fig. 2:**
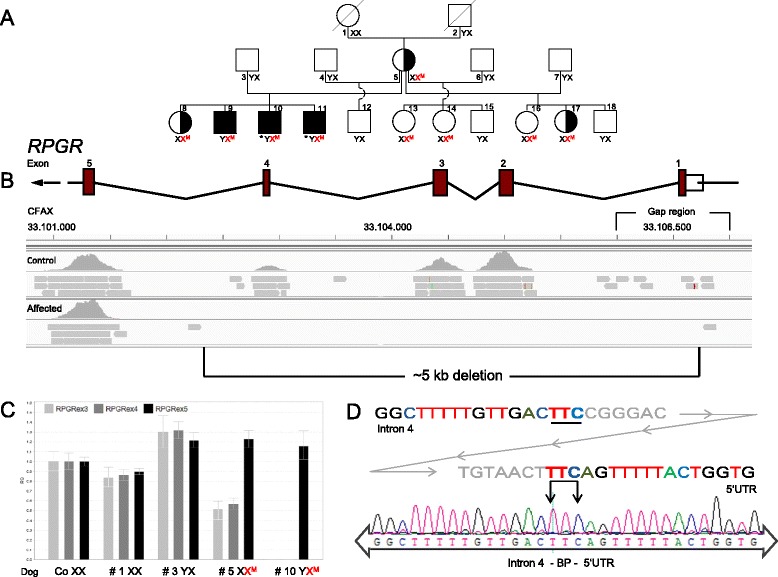
The deletion in the X chromosomal *RPGR* gene as identified in a PRA pedigree of Weimaraner dogs via whole exome sequencing; the breakpoint (BP) region is indicated. **a** Pedigree structure and *RPGR* deletion genotypes of 18 investigated individuals of the XLPRA Weimaraner family. PRA segregates in two generations of the family. Squares represent males, circles indicate females, crossed-out symbols represent deceased dogs. Filled squares show ophthalmologically diagnosed PRA-affected male dogs. Half-filled circles indicate females with ophthalmologically confirmed mild PRA symptoms. Open symbols represent male and female dogs with normal sight as revealed by general veterinarian examinations, respectively. An asterisk below solid square symbols indicates PRA-diagnosed dogs, which were used for whole exome sequencing. Genotypes of *RPGR* deletion screening are shown below the symbols. X^M^ (colored in red) refers to an allele with *RPGR* deletion, X and Y symbols illustrate normal X- (with wildtype *RPGR* alleles) and Y-chromosomes, respectively. **b** Integrated Genomics Viewer (IGV) display of the canine *RPGR* deletion and surrounding regions (CFAX: 3310100–33106500, CanFam3.1 UCSC genome browser) as well as graphical illustration of exon-intron boundaries from the 5′UTR to exon 5. As viewed in IGV, the control and male PRA-affected dog are represented by two separate panels. The upper panel is a histogram where the height of each mountain-like grey area is representative of the read depth at that location. The lower panel is a graphical view of some of the reads that align to that location. Lack of reads (horizontal bars in lower panel) is characteristic for complete loss of exonic sequences. The deletion comprising exons 1–4 (~5 kb) is obvious in the male PRA-affected dog in hemizygous state in contrast to the PRA-unaffected dog. Thus the gap region includes exon 1 in the canine genomic *RPGR* sequence explaining only non-specific read alignments in the lower panel for the control. **c** QPCR-based copy number analysis of the deleted exons 3–4 and the non-deleted exon 5 of *RPGR* gene in four individuals of the pedigree of Weimaraners in comparison to a healthy control. Error bars indicate the standard deviation of three replicates. **d** Chromatogram and graphical representation of the BP region in the *RPGR* gene in a male PRA-affected Weimaraner. The graphical illustration indicates part of intron 4 sequence and of 5′UTR of *RPGR*. Deleted sequences of intron 4 and 5′UTR are coloured in light grey, non-deleted sequences are indicated by coloured letters. The BP region comprises three nucleotides (TTC) from either end which are underlined. The chromatogram also shows the BP (marked with arrows) as well as flanking sequences of intron 4 and 5′UTR of *RPGR* as identified in a male PRA-affected Weimaraner

For whole exome sequencing, we used the genomic DNA of four male Weimaraners, two of them diagnosed with PRA and two healthy control dogs older than the early age of onset (~2.5 years) of this PRA form in Weimaraner dogs. To verify co-segregation, identified variants were investigated in both PRA-affected dogs and their parents by Sanger sequencing. For extended segregation analysis, a pedigree was available comprising 18 Weimaraners, including the two male dogs diagnosed with PRA, their parents, maternal grandparents and two siblings as well as three further breeding partners of the mother and seven half-siblings (Fig. 2a). A cohort of 88 healthy control Weimaraners (41 male, 47 female) which were unrelated to the PRA-affected line for at least five generations was used for validation purposes in case of co-segregation of putative disease causing variants and structural abnormalities using restriction fragment length polymorphism (RFLP) and quantitative real-time PCR (qPCR) analyses, respectively. 90 % of these 88 control dogs were born at least 10 years ago, the youngest male control dogs are 8 and 9 years old by now, respectively. With an average life expectancy of about 12 years, most of the controls are already deceased today. None of the male (and female) control dogs had been reported to the DNA biobank registry as showing restrictions in the visual system to date. All potentially inherited medical impairments are registered in the DNA biobank files of the respective dogs.

### NGS-based mutation screening

Whole exome sequencing was performed on four Weimaraner DNAs (including two of the dogs diagnosed with PRA). The canine exome library consisted of a custom track ordered from Agilent (Santa Clara, CA, USA) as a 54 Mb design. Therefore, tracks of the UC Santa Cruz Genomics Institute (UCSC) Genome Browser from the dog (*Canis familiaris*) whole genome shotgun (WGS) assembly v.2.0 (CanFam2 May 2005) were used as references for the Ensembl Genome Browser (http://www.ensembl.org/index.html) as well as tracks of the National Center for Biotechnology Information (NCBI) reference sequence database (https://www.ncbi.nlm.nih.gov/refseq/rsg/), human protein alignments and spliced expressed sequence tags (EST) that lie outside the tracks of the Ensembl Genome Browser. Sequencing was performed on an Ilumina HiSeq 2000 platform (Illumina Inc., San Diego, CA) at the Cologne Center for Genomics (CCG, Köln, Germany) using a single lane per sample, generating paired-end reads of 2x100 nucleotides (nt) in length and yielding an average coverage of ~60x. Fastq files were further processed using the NextGENe® software (Softgenetics, State College, PA, USA) according to the manufacturer’s protocol. Sequences were aligned to the available reference tracks CanFam2 and xenoRefGene.txt corresponding to the best blast alignment of known genes from other species (available from UCSC Genome Browser, http://hgdownload.cse.ucsc.edu/goldenPath/canFam2/database/) using standard settings. 99.1 % of the reads matched to the reference genome with 82.9 % matching perfectly.

Mutations were called using the NextGENe® software standard settings for a retinal candidate gene panel as well as for the entire exome. For initial retinal candidate gene analysis, a panel of all known RP-associated and retina-specific genes was created based on retinal candidate genes listed by Kastner *et al.* [13] and complemented by candidate genes from the Retinal Information Network (RetNet, http://www.sph.uth.tmc.edu/retnet/) database as well as from recent literature. Since the mode of inheritance of PRA in the Weimaraner breed was not obvious initially, retinal candidate genes were included with different modes of inheritance (autosomal dominant, autosomal recessive and X-linked). Candidate variants were identified by variant comparison and copy number variation (CNV) tools integrated in the NextGENe® software. For variant comparison, PRA-affected individuals were compared to unaffected individuals assuming recessive and dominant modes of inheritance—including the possibility of a heterozygous carrier status in the unaffected dogs in case of recessive inheritance. For CNV analyses, PRA-affected individuals were compared to unaffected individuals using the multiple controls option and the best match mode.

Variations or structural aberrations were considered as candidates for further analyses if they were present at least in heterozygous state in both affected individuals but not in the unaffected ones (dominant model) or if they were present in homozygous state in the PRA-affected dogs and absent or in heterozygous state in the unaffected ones (recessive model). Potential mutations were evaluated in more detail by visualization of the bam-files (binary version of sequence alignment map) with the integrative genomics viewer (IGV; [14]). Diverse online tools and genome browsers, including UCSC Genome Browser (http://genome.cse.ucsc.edu/), Ensembl, Online Mendelian Inheritance in Man (OMIM, http://www.ncbi.nlm.nih.gov/omim), RetNet and GeneCards (http://www.genecards.org/) were also used to provide details about conservation, gene expression, gene function and relevant pathogenic information in man.

### Validation of candidate variants and structural abnormalities

Sanger DNA sequencing of the entire mitochondrial genome (mtDNA) and potential candidate single nt variants was carried out using the Big Dye Terminator kit v3.1 on an ABI 3500XL Genetic Analyzer (Applied Biosystems, Darmstadt, Germany), as previously described [15]. Sequences were evaluated with the Variant Reporter software (Applied Biosystems) and the SeqManII software (DNAstar, Madison, WI, USA). For further validation of potential candidate variants, RFLP analyses were performed as previously described [15]. For verification of structural abnormalities, qPCR was run on the StepOnePlus real time-PCR system (Applied Biosystems) using the KAPA SYBR® FAST ABI Prism™ One-Step qRT-PCR reagent (peqLab, Erlangen, Germany) as described by the manufacturer. Baseline and threshold values were set automatically and cycle threshold (CT) values were determined using the StepOnePlus software (Applied Biosystems). Gene copy numbers were calculated using the ΔΔCT method [16] with normalization to the X-chromosomal dystrophin (*DMD)* gene. Primer sequences used for qPCR are listed in the Additional file 1.

Primers used for candidate gene and mtDNA sequencing, RFLP, qPCR analyses and breakpoint PCR were designed with the LightScanner Primer Design Software v1.0 (Idaho Technology Inc., Salt Lake City, Utah, USA) according to the CanFam2 genome sequence with minimization of possible single nucleotide polymorphisms (SNPs) at the primer binding sites.

Parentage testing was performed using the microsatellite marker based parentage testing kit Canine Genotypes Panel 2.1 on the ABI 3500XL Genetic Analyzer (Applied Biosystems) according to the manufacturer’s protocol. For allele sizing, the GeneScan™ 600 LIZ® Size Standard v2.0 (Thermo Fisher Scientific, Dreieich, Germany) was used. Raw data of microsatellite marker testing were automatically analysed by the ABI 3500XL Genetic Analyzer (Applied Biosystems) and evaluated using the GeneMapper v4.1 software (Applied Biosystems). To confirm the results of the parentage testing kit Canine Genotypes Panel 2.1, DNA profiling was also performed with parentage testing kit Canine Genotypes Panel 1.1 (Thermo Fisher Scientific) which is licensed for parentage testing by the International Society for Animal Genetics.

### *RPGR* genomic sequence and mutation analyses

The canine genomic *RPGR* sequences (chrX: 33056371–33105037, CanFam3.1) were compared to an *in silico*-predicted *RPGR* mRNA in humans (UCSC genome browser accession NM_000328.2) in order to verify exon-intron boundaries. Since the genomic database sequence includes a gap comprising the 5′UTR, exon 1 with the start codon and parts of intron 1 in the canine *RPGR* gene, the respective region was amplified by long-range PCR (expand high fidelity PCR system; Roche, Mannheim, Germany) and sequenced with the Sanger method. The newly identified sequences of the *RPGR* gap region were verified by comparisons with the NCBI database using the Basic Local Alignment Search Tool (BLAST, http://blast.ncbi.nlm.nih.gov/Blast.cgi).

For deletion size estimation in *RPGR*, the newly identified 5′ untranslated region (5′UTR) and part of intron 4 (IVS4) were amplified by PCR using different primer combinations and sequenced with the Sanger method in order to identify the recombination breakpoints. One primer pair was used for breakpoint PCR (diagnostic test) in order to verify qPCR-based deletion screening results. Primer sequences used for recombination breakpoint analysis are listed in the Additional file 1.

## Results

### Phenotype of PRA in Weimaraner dogs

Three male German Weimaraner litter mates were suspected to be affected by PRA upon ophthalmoscopic examination. They showed a significant progressive night blindness with slightly unsafe behaviour in twilight because of visual disturbances. Other than in healthy Weimaraners (Fig. 1a), ophthalmological investigations revealed PRA-like abnormalities such as diffuse hyperreflectivity in the *tapetum ludidum*, depigmentation in the non-tapetal part of the fundus and generalised vascular attenuation (Fig. 1b). All three PRA-affected males were ~2.5 years of age at the time of diagnosis.

Three female Weimaraner dogs (including the mother of the PRA-affected litter mates, one sibling and one half-sibling) showed mild symptoms of PRA at ophthalmological investigations such as patchy and diffuse areas of hypo- and hyperreflectivity in the dorsal *tapetum ludidum*, whereas retinal vessels and non-tapetal fundus were not altered (data not shown). No other symptoms of PRA were found in the female sibling and the mother aged ~2.5 years and ~8 years at diagnosis, respectively. However, the mother was only investigated after the PRA diagnosis in her offspring. For the female half-sibling, ~5 years of age at diagnosis, the owners had noticed slightest changes in visual capability. Progression of retinal degeneration was not documented within the limited time span since PRA was initially diagnosed in the Weimaraner (March to December 2015).

### Candidate gene and mtDNA screening

Due to the unknown inheritance mode of the PRA form in the Weimaraner breed and in order to test the hypothesis of non-mendelian inheritance, the mitochondrial genome (16,727 nt) was analysed for one PRA-affected Weimaraner and his mother. This analysis revealed identical mtDNA haplotypes (the matrilineage) in 100 homoplasmic and eight heteroplasmic variations in both individuals and no differences to healthy dogs in the NCBI database. Thus, no mutation associated with PRA was obvious (data not shown).

### Next generation and Sanger sequencing

Whole exome sequencing was performed in two male PRA-affected and two male healthy control Weimaraner dogs. In total, 351,538 sequence variants were identified. Initial retinal candidate gene panel filtering for 346 genes (see Additional file 1) causing autosomal dominant, recessive or X-linked inherited retinal diseases revealed no PRA causing mutation for the PRA-affected dogs. Therefore, comparison analyses of all 351,538 whole exome sequence variants of PRA-affected *versus* control dogs assuming recessive or dominant inheritance were performed by using the comparison tool of the NextGENe® software. In total, 6,805 potentially recessive and 15,135 potentially dominant variants were identified. Most variants were excluded as causative for PRA due to information concerning poorly conserved locations, non-retina-specific gene expression and function from diverse public databases. However, four potential variants (two nonsense and two missense mutations) in the genes endoplasmic reticulum aminopeptidase 2 (*ERAP2*), DnaJ/Hsp40 homolog, subfamily c, member 4 (*DNAJC4*), exportin 4 (*XPO4*) and dihydropyrimidine dehydrogenase (*DPYD*) were not excluded by the aforementioned criteria and subsequently verified by Sanger sequencing. Analyses of these four sequence variants demonstrated segregation in both PRA-affected dogs and their parents. However, RFLP analyses of a cohort of 88 unrelated control Weimaraners excluded all four variants as causative for PRA since they were identified in numerous healthy individuals. Next, comparisons of whole exome CNVs of PRA-affected *versus* control Weimaraners were performed by using the CNV tool of the NextGENe® software. In total, 341 deletions and 552 duplications were identified and verified with the IGV viewer. Among these, a large deletion of exon 1–4 of the X-linked *RPGR* gene was identified in the PRA-affected Weimaraners, which was not found in the controls (Fig. 2b). *RPGR* is a PRA candidate gene (RetNet database) since mutations in this gene have been described to cause both XLRP in man [11] and X-linked PRA in dogs [7].

### Mutation screening

The presence of the *RPGR* deletion was verified in both male PRA-affected dogs (by qPCR analysis of the deleted exons 3–4 and the non-deleted exon 5) in comparison to their parents (#3, #5), their maternal grandmother (#1) and a healthy control. Compared to the control with normal copy number for all three exons, the PRA-affected dog (#10) lacked these exons, and his mother (#5) showed just one copy for exons 3 and 4, while two copies for exon 5 were identified in both of them. For all three exons, two copies were evidenced in his grandmother (#1) and one copy in his father (#3; Fig. 2c). QPCR-based screening of the deleted exon 4 in 13 further individuals of the pedigree of the affected dogs revealed no copy for another male sibling (#9) and one copy for another female sibling (#8) as well as four female half-siblings (#13–14, #16–17). Two copies of exon 4 were shown for the maternal grandfather (#2), three male half-siblings (#12, #15, #18) and the three breeding partners of the mother (#4, #6–7). In total, qPCR analyses verified the deletion in the three affected males and the three mildly affected females in the investigated Weimaraner pedigree (Fig. 2a). Additionally, the deletion was demonstrated in three female dogs which so far have not revealed any clinical symptoms. In 88 unrelated control Weimaraners, the presence of exon 4 was proven by normal copy numbers (data not shown). After identification of *RPGR* deletion breakpoints, a breakpoint PCR (diagnostic test) was performed for all 18 individuals of the pedigree confirming the results of *RPGR* deletion screening as identified by qPCR and indicating that the deletion comprises exons 1–4 of the *RPGR* gene (data not shown).

In order to verify that the X-linked PRA in the investigated Weimaraner pedigree is caused by a *de novo* mutation, we investigated the biological parentage of both the mother and the maternal grandparents of the PRA-affected dogs by using the microsatellite marker based parentage testing kits Canine Genotypes Panel 2.1 and Canine Genotypes Panel 1.1. No inconsistencies concerning the ancestry were identified in the investigated individuals.

### Mutation detection

In order to determine the genomic size of the *RPGR* deletion, the recombination breakpoints in IVS4 and 5′UTR were analysed by PCR amplification employing various primer sets. However, via UCSC database comparisons, a gap region was identified in the canine *RPGR* sequence comprising 5′UTR, exon 1 with the start codon and parts of intron 1. The sequence of this gap was analysed by long-range PCR and subsequent primer walking. The newly identified DNA sequence of this gap region comprised 846 nt including a known long interspersed nuclear element (LINE, repeat L1_Canis1_family L1, UCSC genome browser) which occurs several times in the canine genome (NCBI GenBank accession no KU234669). A part of the newly identified genomic sequence of exon 1 was also detected in an mRNA isoform of the *RPGR* gene in *Canis lupus familiaris* (accession NM_001003126.1). As indicated by sequencing, the breakpoint regions comprised three nucleotides (TTC, Fig. 2d). The exact breakpoints are localized in the newly identified 5′ sequence of *RPGR* gene (chrX: 33106747 + 190, UCSC genome browser, CanFam3.1, September 2011 assembly) and in IVS4 (chrX: 33102324), respectively. Thus, the *RPGR* deletion comprised a maximum size of 5,006 nt in the mutation-carrying Weimaraners.

## Discussion

In the spring of 2015, ophthalmological examinations had revealed retinopathy in two male symptomatic Weimaraners from a single litter born in 2012. Female dogs of this and another maternal litter were diagnosed with milder impairments. The veterinarians (at DOK) named this form of retinal disease in Weimaraner retinopathy instead of PRA, because clear progression was not documentable within the limited time span. Nonetheless, since deleterious mutations in the *RPGR* gene cause XLPRA, we use the original designation.

Here, whole exome sequencing was applied to map the causative mutation for this early-onset form of PRA in Weimaraners. Several litter mates had been diagnosed with PRA, but the mode of inheritance was not clear initially. One cause for RP in man are matrilineally inherited mutations in the mitochondrial genome [17]. Analysis of the latter revealed no obvious pathogenic differences in Weimaraners.

Using NGS-based whole exome analysis, a large deletion comprising the first four exons including the start codon was identified in the X-chromosomal *RPGR* gene in PRA-affected Weimaraners. This gene encodes a binding protein with a N-terminal domain homologous to the regulator of chromosome condensation 1 (*RCC1*), a guanine nucleotide exchange factor for the small GTPase Ran [18], which is important for the association with binding partners [19]. RPGR transcripts undergo extensive alternative splicing *e.g.* in man, mouse and dog [20, 21] producing constitutive and open reading frame 15 (ORF15) variants with an additional exon ORF15 by using alternative polyadenylation and splice sites [22]. The constitutive variant is expressed in a wide variety of tissues [20], whereas the ORF15 variant is predominantly found in the retina [22]. The retinal subcellular localization of RPGR protein is shown to be relatively consistent across several mammalian species with high enrichment in the photoreceptor connecting cilium [23]. Although the exact function of RPGR is not fully understood, studies suggest that it plays an important role in photoreceptor survival and its development [7]. RPGR has been shown to interact with a variety of ciliary proteins such as RPGRIP1 [24]. In this context six candidate genes were discussed as potential modifiers in canine XLPRA [25]. Mutations in the *RPGR* gene account for over 70 % of X-linked RP [22, 26] and 15–20 % of all RP [27]. Exon ORF15 is known to be a mutational hotspot for XLRP, accounting for about 70 % of all disease-causing mutations in the *RPGR* gene in humans [28]. Two canine animal models of ORF15 frame-shift mutations have been published which resemble the human phenotype in disease onset and progression [7, 21]. Thus, the Weimaraner breed is the third naturally occurring pure-bred animal model for XLRP in addition to Siberian husky and Samoyed [7, 21]. However, it is the first one with a causative mutation localized in the highly conserved 5′ terminus of the *RPGR* gene in contrast to the mutational hotspot in exon ORF15. Since the identified *RPGR* deletion was not detectable in the maternal grandparents of the affected male Weimaraners, biological parentage had to be confirmed by microsatellite marker testing. The deletion was also absent in 88 unrelated Weimaraner dogs, the deletion has occurred *de novo*.

Based on comparisons with the location of CpG islands in the human *RPGR* reference sequence (UCSC genome browser, hg38, December 2013 assembly), we suggest that the canine *RPGR* promotor region is situated in the newly identified DNA sequence in the 5′ sequence of the gene, outside of the 5′terminal breakpoint region. Therefore, the deletion comprised the first four exons including the start codon of the *RPGR* gene, suggesting that the translation initiation is impaired resulting in the lack of *RPGR* gene products. Previous analyses of mice lacking *Rpgr* showed that photoreceptor morphology was initially normal but photoreceptor cell degeneration was noted over time, suggesting that the *Rpgr* gene is required for long-term maintenance of photoreceptors [29]. This correlates with the PRA form studied here in Weimaraners which presented with initial PRA symptoms at ~2.5 years of age. In addition, it has been shown that a 2 nt- frameshift microdeletion causes photoreceptor degeneration in canine X-linked PRA [30]. Finally, UCSC Genome Browser comparisons with human *RPGR* transcripts demonstrate that a functional transcript lacking the first four exons has not been observed so far.

Our molecular genetic results of the *RPGR* deletion as evidenced in the Weimaraner pedigree studied (Fig. 2a) were entirely consistent with the respective results of ophthalmological investigations by DOK veterinarians. The deletion was identified in hemizygous state in three male Weimaraner litter mates all of which were ophthalmologically diagnosed with PRA and showed diffuse tapetal hyperreflectivity, attenuated vessels (Fig. 1b) and visual disturbances in twilight compared to a dog with normal central fundus (Fig 1a) and without visual impairments. In six female individuals of the pedigree the deletion was found in heterozygous state, and ophthalmological investigations in three of them revealed mild symptoms of peripheral hyperreflexia (data not shown). These findings are in accordance with a recessive mode of X-linked inheritance in which hemizygous males are usually severely affected, while female individuals carrying the deletion in heterozygous state may be asymptomatic or, in case of non-random or skewed X-inactivation, may present a mild phenotype [31].

## Conclusions

The findings of the present study demonstrate the value of an existing DNA biobank combined with modern tools available for genomic mutation analyses to aid rapid identification of the genetic cause of a spontaneously occurring disease in a dog breed. Without the DNA biobank for Weimaraner dogs, it would have been impossible to include the deceased maternal grandparents in this study. This implies that the *RPGR* deletion screening would have had to be performed in a much larger population of Weimaraners. In short, a diagnostic test has been established, that allows for mutation screening for both very young and (still) healthy dogs. Thus, precise conclusions may be drawn, and the PRA mutation may be eradicated from the respective Weimaraner line.

## Abbreviations

5′UTR, 5′ untranslated region; BLAST, basic local alignment search tool; BP, breakpoint; CCG, cologne center for genomics; CNV, copy number variation; CT, cycle threshold; *DMD*, dystrophin; *DNAJC4*, DnaJ/Hsp40 homolog, subfamily c, member 4; DOK, *Dortmunder Kreis*; *DPYD*, dihydropyrimidine dehydrogenase; *ERAP2*, endoplasmic reticulum aminopeptidase 2; EST, expressed sequence tags; IGV, integrative genomics viewer; IVS4, intron 4; LINE, long interspersed nuclear element; mtDNA, mitochondrial genome; NCBI, national center for biotechnology information; NGS, next generation sequencing; nt, nucleotides; OMIM, online mendelian inheritance in Man; ORF15, open reading frame 15; PRA, progressive retinal atrophy; qPCR, quantitative real-time PCR; *RCC1*, regulator of chromosome condensation 1; RetNet, retinal information network; RFLP, restriction fragment length polymorphism; RP, retinitis pigmentosa; *RPGR*, retinitis pigmentosa GTPase regulator; SNPs, single nucleotide polymorphisms; UCSC, UC Santa Cruz Genomics Institute; VDH, *Verband für das Deutsche Hundewesen*; WGS, whole genome shotgun; XLRP, X-linked retinitis pigmentosa; *XPO4*, exportin 4

## Additional file

Additional file 1:
**A** Candidate genes for PRA exome screening (*n* = 346). **B** and **C** Primers used for breakpoint analysis (**B**) and qPCR (**C**) with their corresponding product size. (XLSX 19 kb)
